# Efficacy of Sodium-Glucose Cotransporter-2 inhibitors in heart failure patients treated with dual angiotensin receptor blocker-neprilysin inhibitor: An updated meta-analysis

**DOI:** 10.1016/j.amsu.2021.102796

**Published:** 2021-09-08

**Authors:** Naser Yamani, Fahd Niaz Shaikh, Saba Sarfraz, Haider Kamal Khan, Muhammad Fahad Wasim, Anousheh Awais Paracha, Talal Almas, Farouk Mookadam, Samuel Unzek

**Affiliations:** a Department of Medicine, John H Stroger Jr. Hospital of Cook County, Chicago, IL, USA; b Department of Medicine, Dow University of Health Sciences, Karachi, Pakistan; c Department of Medicine, Islamabad Medical and Dental College, Islamabad, Pakistan; d Department of Medicine, Baqai Medical University, Karachi, Pakistan; e Department of Medicine, RCSI University of Medicine and Health Sciences, Dublin, Ireland; f Director of Cardio Oncology, Banner University Medical Centre, Phoenix, AZ, USA; g Director of Cardiac Imaging, Banner University Medical Centre, Scottsdale, AZ, USA

**Keywords:** Sodium-Glucose Cotransporter-2 inhibitors, Dual angiotensin receptor blocker-neprilysin inhibitor, Heart failure, Meta-analysis

## Abstract

**Background:**

Recent data suggest that the prevalence of heart failure has increased to approximately 23 million people globally. With increasing advancement in pharmacotherapeutics, Sodium-Glucose Cotransporter-2 inhibitors (SGLT2i) have garnered attention among clinicians to treat Heart failure with reduced ejection fraction (HFrEF) in diabetic as well as non-diabetic patients.

**Methods:**

MEDLINE, Scopus, Embase and Cochrane CENTRAL database were searched using relevant keywords and MeSH terms. Studies were considered only if they were randomized in nature and had a sample size >1000 HF patients.

**Results:**

Our comprehensive search strategy yielded 864 articles, of which three RCTs met the inclusion criteria with a total population of 9696. Pooled analysis revealed an association between the use of SGLT2i and decreased frequency of primary outcome irrespective of background ARNI use (HR 0.73, 95% CI [0.58–0.93], p = 0.0106; HR 0.73, 95% CI [0.66–0.81], p < 0.0001).

**Conclusion:**

This meta-analysis provides substantial evidence, to safely use SGLT2i atop ARNI therapy in select HF patients to further improve outcomes.

## Highlights

• Heart failure (HF) prevalence has increased to approximately 23 million people globally.
• With advancements in treatment strategies for HF patients, a novel drug class, SGLT2i has garnered attention among clinicians.
• This meta-analysis collects the latest trial-based evidence and confirms the association between decreased frequency of primary outcome and SGLT2i use.

## 1 Introduction

Recent data suggest that the prevalence of heart failure has increased to approximately 23 million people globally, with half of the cases contributed by heart failure (HF) with preserved ejection fraction (HFpEF) [1]. With the recent rapid progress in pharmacotherapeutics, there have been advancements in the management and treatment of HF. Previously, dual angiotensin II receptor blockers-neprilysin inhibitor (ARNI) have been proven to treat HF patients successfully. More recently, Sodium-Glucose Cotransporter-2 Inhibitors (SGLT2i) have garnered attention among clinicians managing patients with congestive heart failure, because of the clinical trials using SGLT2i show an important reduction in mortality, morbidity, and adverse renal outcomes in diabetic and non-diabetic patients with Heart failure with reduced ejection fraction (HFrEF) [2]. In this meta-analysis, we aimed to collect evidence from all available RCTs and pooled their results to assess the risks and benefits of this drug class.

## 2 Materials and methods

A comprehensive electronic literature search was performed, using databases from MEDLINE, Scopus, Embase and Cochrane CENTRAL from inception to February 28th, 2021. Two independent reviewers (MFW and AAP) then individually extracted studies that met our inclusion criteria based on titles and abstracts. Any disagreement regarding study selection was resolved by discussion with a senior investigator (FM). Studies were selected for full-text appraisal if they matched predefined eligibility criteria of: (1) randomized controlled trials (RCTs) with SGLT2i placebo in two arms, (2) population sample size >1000 HF patients, (3) articles published in the English language. The articles retrieved were compiled in Endnote Reference Library (Version X7.5; Clarivate Analytics, Philadelphia, Pennsylvania) software and matching articles were removed. All remaining articles were then reviewed to ensure that they met the predefined eligibility criteria. The primary outcome of our analysis was the combined incidence of cardiovascular (CV) death, hospitalizations related to HF, which were then analyzed using Hazard Ratios (HR). Using Cox proportional hazard models, HRs and their 95% confidence intervals (CIs) were converted to their natural logarithm [ln (HR)] and corresponding SEs. We pooled these values for the meta-analysis. Heterogeneity in effect sizes was estimated using Higgin's I^***2***^ statistics, where the I^***2***^ value > 50% was considered significant. We also performed the Egger's regression test and used funnel plots to check for any publication irregularities. The meta-analysis was performed using RevMan (version 5.3; Copenhagen: The Nordic Cochrane Centre, The Cochrane Collaboration). Reporting quality was evaluated using Preferred Reporting Items for Systematic Reviews and Meta-Analyses (PRISMA) [3] and methodological quality using the Assessment of Multiple Systematic Reviews (AMSTAR-2) tool [4].

## 3 Results and discussion

**Fig. 1:**
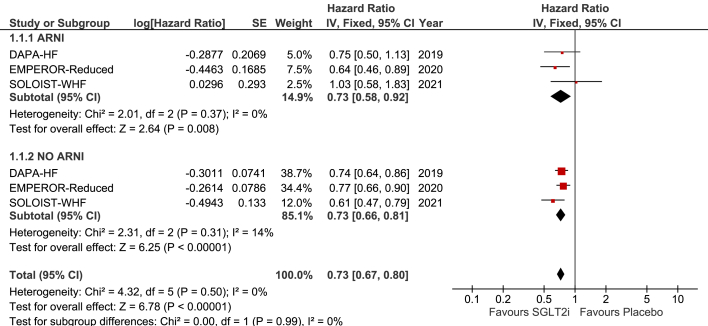
Forest plots displaying the incidence of the primary outcome. Figure (1.1.1) shows subgroup results for patients treated with combined therapy of SGLT2i and ARNI. Figure (1.1.2) shows subgroup results for patients treated with SGLT2i only. (CI: confidence Interval; IV: inverse variance; SE: standard error).

Our comprehensive search strategy yielded 864 articles, of which three RCTs (DAPA-HF, EMPEROR-Reduced, and SOLOIST-WHF), met the inclusion criteria with a total population of 9696. Study characteristics are summarized in Table 1. Our meta-analysis revealed an association between the use of SGLT2i and decreased frequency of primary outcome irrespective of background ARNI use (HR 0.73, 95% CI [0.58–0.93], p = 0.0106; HR 0.73, 95% CI [0.66–0.81], p < 0.0001) **(**Fig. 1**)**. Statistical heterogeneity was found to be non-significant (p > 0.05). Considering these results, we can conclude that SGLT2i has therapeutical benefit in HF patients even in the absence of ARNI use.

**Table 1:** Baseline and study characteristics of included studies.

Study name, year of publication	Study design	Drug Compound	Sample size(n)	Follow up (days)	Age (median years)	Female (%)	Diabetes Mellitus (%)	*n*-proBNP, median pg/mL	ARNI (%)	Renin-angiotensin inhibitors without neprilysin inhibitor (%)	Beta-Blockers (%)
DAPA-HF (2019)	Randomized controlled trial	Dapagliflozin	4744	730	dapagliflozin = 66	dapagliflozin = 23.8	dapagliflozin = 41.8	dapagliflozin = 1428.0	dapagliflozin = 10.5	dapagliflozin = 84.5	dapagliflozin = 96.0
			dapagliflozin = 2373		placebo = 66	placebo = 23.0	placebo = 41.8	placebo = 1446.0	placebo = 10.9	placebo = 82.8	placebo = 96.2
			placebo = 2371								
EMPEROR-Reduced (2020)	Randomized controlled trial	Empagliflozin	3730	810	empagliflozin = 67	empagliflozin = 23.5	empagliflozin = 49.8	empagliflozin = 1887.0	empagliflozin = 18.3	empagliflozin = 70.5	empagliflozin = 94.7
			empagliflozin = 1863		placebo = 66	placebo = 24.4	placebo = 49.8	placebo = 1926.0	placebo = 20.7	placebo = 68.9	placebo = 94.7
			placebo = 1867								
SOLOIST-WHF (2021)	Randomized controlled trial	Sotaglifozin	1222	274	empagliflozin = 69	empagliflozin = 32.6	empagliflozin = 12.7	empagliflozin = 1816.8	empagliflozin = 15.3	empagliflozin = 82.1	empagliflozin = 92.8
			sotaglifozin = 608		placebo = 70	placebo = 34.9	placebo = 11.1	placebo 1741.0	placebo = 18.2	placebo = 83.3	placebo = 91.4
			non-statin = 614								

(ARNI: Angiotensin neprilysin inhibitors; *n*-proBNP: *N*-terminal prohormone of brain natriuretic peptide).

The initial therapeutic roles of the ARNI and SGLT2i differed vastly. The SGLT2i drug class are long known reliable and effective, diabetic therapy in contrast to ARNI, which is used singularly for the treatment of chronic heart failure with reduced ejection fraction. The pivotal trials conducted thus far [[5], [6], [7]], demonstrate, cardioprotective mechanisms against HF. When Zannad et al. pooled the data from these RCTs, a subgroup analysis showed better outcomes in pre-existing ARNI users as well as those were ARNI naïve and on an SGTL2i [8]. These results serve as an encouragement for researchers and can be hypothesis-generating for future studies to confirm this correlation sought to investigate the benefits of SGLT2i in patients with heart failure, regardless of background guideline directed medical therapy (GDMT).

This meta-analysis is subject to a few limitations. First, the designs of the aforementioned trials were not made to establish the superiority of one drug class over the other. Second, our analysis was limited to study-level data rather than patient-level data leading to potential associations not being investigated. Third, it should be acknowledged that despite consistent results, the heterogeneity cannot be reliably interpreted if the statistical analysis is computed using only three studies. Moreover, SOLOIST-WHF trial itself presents some limitations. The patient population was subject to a lack of diversity in age, diabetes type and suffered from deteriorating HF symptoms. The majority of patients presented with classes III and IV HF symptoms at baseline. The termination of the trial prematurely leads to shorter follow-up of the patients. Considering these limitations, we believe that future clinical trials are warranted to better understand the outcomes associated with combination therapy in the HF population.

## 4 Conclusion

In conclusion, this meta-analysis provides substantial evidence, to safely use SGLT2i atop ARNI therapy in select HF patients to further improve outcomes.

## Sources of funding

NA.

## Ethical approval

NA.

## Consent

NA.

## Author contributions

Naser Yamani and Fahd Niaz Shaikh conceived the idea and designed the study.

Saba Sarfraz and Haider Kamal Khan drafted the manuscript.

Muhammad Fahad Wasim and Anousheh Awais Paracha conducted the literature review.

Talal Almas, Farouk Mookadam and Samuel Unzek reviewed and finalized the manuscript.

## Trial registry number

1. Name of the registry: NA.

2. Unique Identifying number or registration ID: NA.

3. Hyperlink to your specific registration (must be publicly accessible and will be checked): NA.

## Guarantor

Fahd Niaz Shaikh.

Dow University of Health Sciences, Karachi.

## Disclosures

NA.

## Provenance and peer review

Not commissioned, externally peer reviewed.

## Declaration of competing interest

None.
